# Microstructure and First Hydrogenation Properties of Zr_1−x_Ti_x_Cr_2_ Alloys Where x = 0, 0.25, 0.5, 0.75, and 1

**DOI:** 10.3390/molecules30173611

**Published:** 2025-09-04

**Authors:** Tanin Bakhtiari, Salma Sleiman, Jacques Huot

**Affiliations:** Hydrogen Research Institute, Université du Québec à Trois-Rivières, 3351 des Forges, Trois-Rivières, QC G9A 5H7, Canada; tanin.bakhtiari@uqtr.ca (T.B.); salma.sleiman@uqtr.ca (S.S.)

**Keywords:** metal hydrides, C14 Laves phase, BCC, Zr substitution, first hydrogenation

## Abstract

Metal hydrides are an attractive way to store hydrogen in a compact and safe manner under low pressure. However, one of the hurdles to the widespread use of this method is the difficulty of the first hydrogenation, which increases the material cost. In this paper, we report the effect of substituting Zr with Ti in Zr_1−x_Ti_x_Cr_2_ alloys (x = 0, 0.25, 0.5, 0.75, and 1) on the first hydrogenation. All the substituted alloys had similar microstructures and crystallized in the metastable C14 Laves phase. For x = 0, the first hydrogenation was possible at room temperature under 2 MPa of hydrogen pressure. As x increased, the hydrogen capacity decreased. For x = 0.75 and 1, first hydrogenation was practically impossible.

## 1. Introduction

In recent years, hydrogen has gained attention as a promising energy carrier due to its high energy efficiency, environmental benefits, and abundance. It has potential applications in portable power devices, on-board fuel cells, and stationary systems. However, industrial adoption of hydrogen has been limited by the lack of safe and efficient storage methods [1,2,3,4]. One of the safer and more compact methods for storing hydrogen is in its atomic form in metal hydrides. Metal hydrides could have a volumetric capacity of hydrogen of up to 150 g/L, which is twice the density of liquid hydrogen (70.8 g/L) [5].

Metal hydrides form through the reversible interaction between hydrogen gas and a hydride-forming metal. Over recent decades, numerous materials have been investigated for hydrogen storage, including Mg [6], alloys, intermetallic compounds such as LaNi_5_ (AB_5_ type) [7], FeTi (AB type) [8], and Laves phases (AB_2_ type). They are classified based on their enthalpy of hydride formation: A-type elements have a more negative enthalpy, indicating a higher tendency to form hydrides, whereas B-type elements have a less negative or even positive enthalpy, indicating a lower affinity for hydrogen [9].

Laves phases (AB_2_) are categorized into three structural types: the hexagonal MgZn_2_ type (C14), cubic MgCu_2_ type (C15), and hexagonal MgNi_2_ type (C36), with C14 being the most common [10]. These structures contain three types of tetrahedral interstitial sites, A_2_B_2_, AB_3_, and B_4_, that vary in their preference for hydrogen absorption. Hydrogen atoms predominantly occupy the A_2_B_2_ sites, followed by AB_3_ sites, while B_4_ sites cannot accommodate hydrogen atoms. Although not all sites can be simultaneously occupied, this variety allows AB_2_ Laves phases to exhibit good hydrogen-absorption properties. Theoretically, the maximum hydrogen capacity of C14-type and C15-type Laves phases is up to 6.3 and 6 hydrogen atoms per formula unit, respectively [11,12].

Among the AB_2_ Laves-type hydrides, ZrCr_2_ forms a stable hydride under ambient conditions [13]. ZrCr_2_ can crystallize in multiple phases: C14 hexagonal MgZn_2_-type (space group P6_3_/mmc), C36 hexagonal MgNi_2_-type (space group P6_3_/mmc), and C15 cubic MgCu_2_-type (space group Fd-3m) phases. The differences between the C14 and C36 phases are their stacking sequences (ABAC for C14 vs. ABACBCAC for C36) and the number of atoms per unit cell (12 for C14 vs. 24 for C36) [14,15,16]. According to the Zr-Cr phase diagram, the C14 phase is stable between 1946 K and 1913 K, the C36 phase between 1913 K and 1846 K, and the C15 phase below 1846 K. These phases transform with temperature changes during heating and cooling [17]. The absorption capacity of ZrCr_2_ at room temperature is either 3.4 hydrogen atoms per formula unit at 0.1 MPa for the C14 type or 4 hydrogen atoms per formula unit at 6.2 MPa for the C15 type [18]. Bodega et al. have shown that the different types of Laves phases could be synthesized by varying the cooling rate during synthesis [19]. They prepared hexagonal samples, predominantly consisting of the C14 and C36 phases with a minor amount of the C15 phase, by alloying the materials at 2200 K. In contrast, C15 samples were synthesized by alloying at 2073 K, followed by annealing at 1540 K for 3 h under vacuum and subsequently cooling them to room temperature. They found that the hydrided hexagonal phases (C14 and C36) are more stable than the C15 cubic phase, as determined by pressure–composition–temperature (PCT) measurements.

In the case of TiCr_2_ alloy, it also exhibits different crystal structures depending on the temperature. The alloy forms the high-temperature C14 phase at 1359 °C. Upon cooling below 1271 °C, the alloy undergoes a phase transition to the intermediate-temperature hexagonal C36 polytype. Further cooling to 1223 °C stabilizes the C15 phase [14,20].

The hydride phase formation of the hexagonal ZrCr_2_ has an enthalpy of −21.0 ± 0.8 kJ (mol H_2_)^−1^ and an entropy of −48 ± 5 J (mol H_2_)^−1^ K^−1^ [19]. In comparison, the C14 phase of TiCr_1.9_ has an enthalpy of −26.5 ± 1 kJ (mol H_2_)^−1^ and an entropy of −122.0 ± 4 J (mol H_2_)^−1^ K^−1^ [21]. These thermodynamic parameters show the significant differences in the stability of hydride formation between the ZrCr_2_ and TiCr_1.9_ phases. By systematically varying x, we aimed to see how the relative stability of these hydride phases changes with Ti substitutions.

Substitution of Zr with Ti was investigated by Bulyk et al. using the HDDR (Hydrogen-Induced Phase Transformation) process [22]. They found that the partial replacement of zirconium with titanium in the ZrCr_2_ intermetallic compound led to an increase in its stability in hydrogen and slowed down the disproportionation reaction. Dovhyi showed that the stability of the crystal structure is determined by the factor of the interaction of the Fermi surface and the Brillouin zone [23]. Klyamkin et al. studied the phase transformations of TiCr_1.8_, ZrCr_2_, Ti_0.9_Zr_0.1_Cr_1.8_, Ti_0.7_Zr_0.3_Cr_1.8_, and TiCr_1.7_Fe_0.1_ alloys under hydrogen at pressures from 0 to 199 MPa and temperatures of −78 °C and 22 °C [24]. They found that all these alloys have a C14 Laves phase structure and the hydride phase has the same structure when the hydrogenation is performed at room temperature.

In this study, we investigated the Zr_1−x_Ti_x_Cr_2_ system with x values of 0, 0.25, 0.5, 0.75, and 1. Substitution of Zr with Ti was chosen due to the miscibility of Ti and Zr in various proportions. Our objective was to examine how these substitutions affect the crystal structure and microstructure as well as the initial hydrogenation behaviour of the resulting Zr_1−x_Ti_x_Cr_2_ alloys.

## 2. Results and Discussion

### 2.1. Microstructural Study

**Figure 1:**
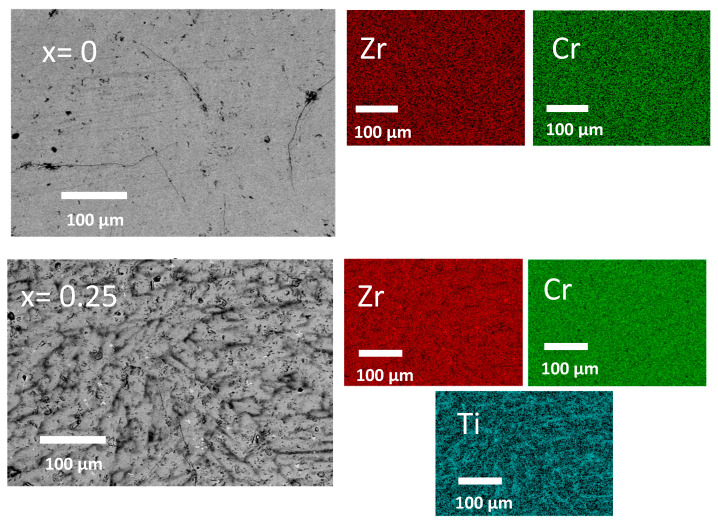

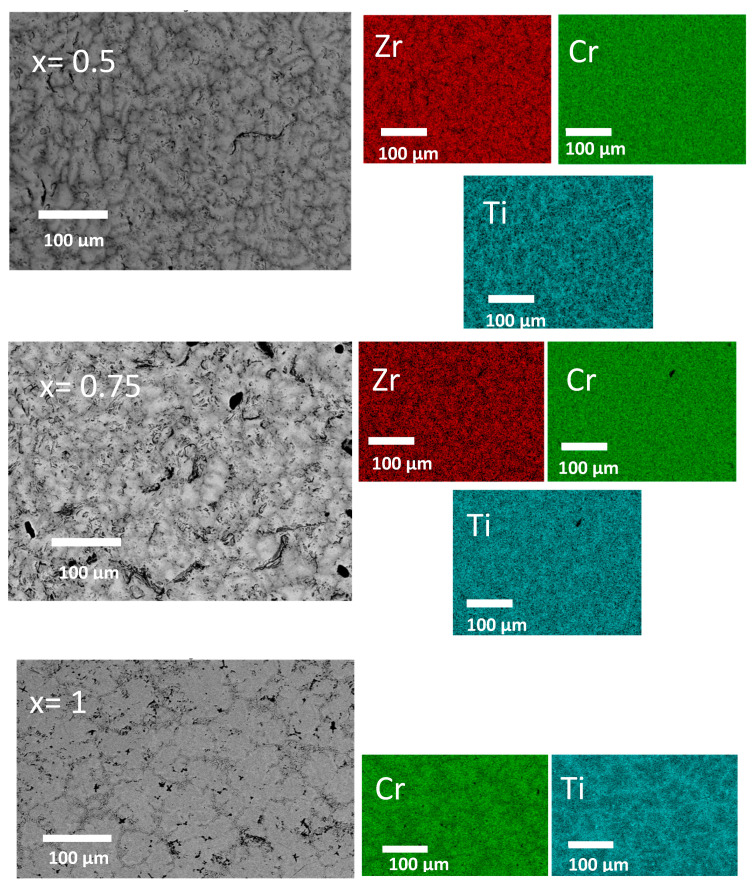
Backscattered electron (BSE) micrograph of Zr_1−x_Ti_x_Cr_2_ alloys where x = 0, 0.25, 0.5, 0.75, and 1 with element mapping.

Figure 1 shows the backscattered electron micrograph and element mapping of Zr_1−x_Ti_x_Cr_2_ alloys where x = 0, 0.25, 0.5, 0.75, and 1. It should be pointed out that no oxygen peak was seen in the EDX spectra of all the compositions. This is an indication that no oxides were formed.

Figure 1 shows that the microstructure changes upon substitution of Zr with Ti. For x = 0, Zr and Cr are uniformly distributed in the alloy. Upon substitution, two slightly different shades of grey regions (“dark and light”) appeared. Cr remains uniformly distributed over the alloy while Ti and Zr seem to be more concentrated in some regions and mutually exclusive. However, the mapping micrographs did not show any region of pure Ti or with a high concentration of Ti. This is an indication that Ti completely replaced the Zr in all the alloys. To check the bulk chemical compositions, EDX measurements were performed on all the alloys. Table 1 shows the bulk-measured atomic abundance compared to the nominal composition. We can see that the bulk-measured composition agrees with the nominal one in all cases.

Using EDX, we also measured the chemical composition of the different grey regions for all the alloys. The results are reported in Table 2.

Table 2 shows that the composition of the dark grey regions has a higher proportion of Ti than their light grey counterparts. The abundance of chromium is the same for all regions. Regarding Ti and Zr abundances, we can see that the sum is always close to 33%, which indicates that both regions should have an AB_2_ structure. As there is a gradual change in the grey intensity between the dark and the light regions, we should not interpret the values in Table 2 as an indication that there are two AB_2_ structures in the alloy with a definite stoichiometry. Instead, it should be seen as a smooth variation in stoichiometry between the two end members, which is reflected by the light and dark region compositions shown in Table 2.

### 2.2. Crystal Structure

**Figure 2:**
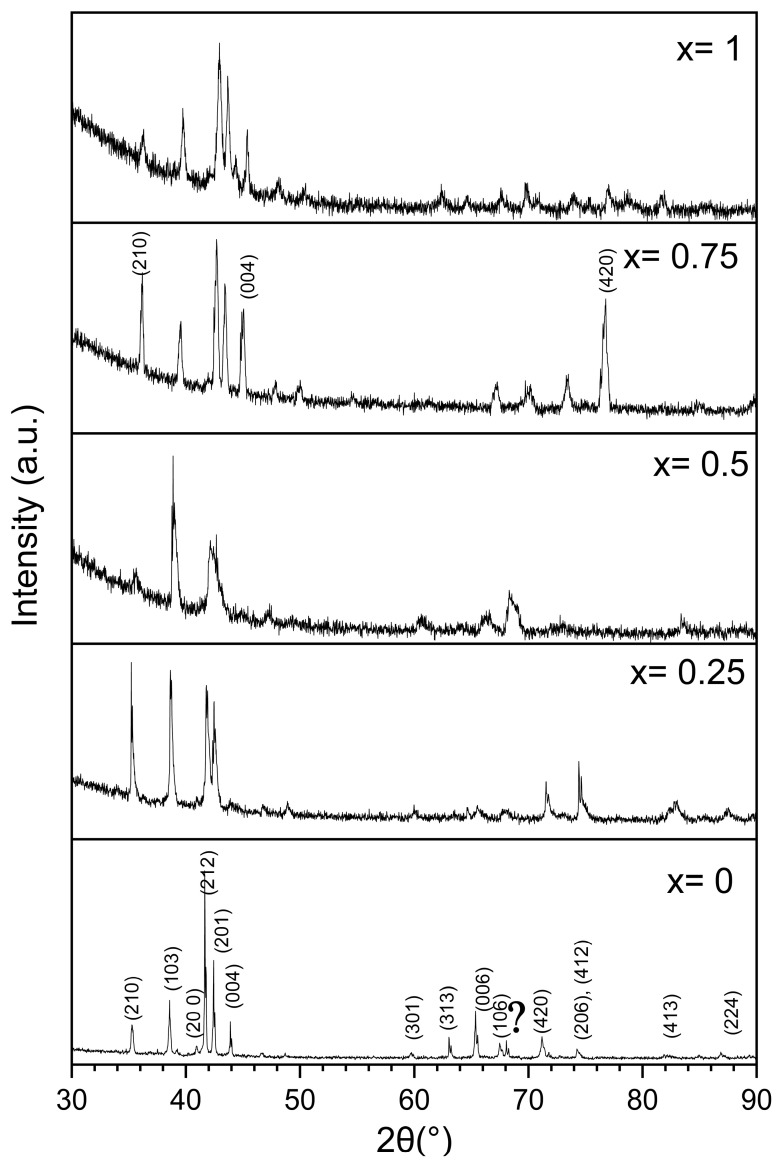
XRD patterns of as-cast Zr_1−x_Ti_x_Cr_2_ alloys where x = 0, 0.25, 0.5, 0.75, and 1. The position and Miller index of the Bragg’s peaks of the C14 phases are shown for the X = 0 pattern. The ? indicates an unknown phase.

The X-ray powder diffraction results for the Zr_1−x_Ti_x_Cr_2_ alloys where x = 0, 0.25, 0.5, 0.75, and 1 are shown in Figure 2.

We can see that the relative intensities of the peaks change greatly with composition. However, Rietveld refinement of these patterns indicated that they are all single-phase C14 except for the TiCr_2_ alloy, which has a small BCC (Body-Centred Cubic, space group Im-3m, prototype W) component (9 wt.%). In this pattern, we can also see small peaks at around 67° that we could not index to any phase. The relative intensities of the end members (TiCr_2_ and ZrCr_2_) closely match the C14 phase. However, for the other compositions, even if the pattern fits the C14 phase, the intensities of some planes do not match the nominal intensities. For example, in the pattern for x = 0.75, the peaks at around 36°, 45°, and 76° are too intense compared to the expected intensities. The Miller indexes of these peaks are, respectively, (210), (004), and (420). This may be an indication that there are some crystallographic planes where the Ti-Zr substitution is not random, which results in the formation of a superstructure. In accordance with Bodega et al. [19], a C36 phase was included in the trial refinement, but it never improved the fit. Therefore, we can conclude from the X-ray powder diffraction patterns that the C36 phase does not form in any of the compositions synthesized in the present investigation.

Based on the phase diagrams of Zr-Cr and Ti-Cr systems, the stable phase expected for ZrCr_2_ and TiCr_2_ alloys at room temperature is the cubic C15 phase [19,24]. However, our experimental findings only detected the hexagonal C14 phase. In light of Bodega et al.’s findings [19], this could be explained by the high temperature reached during arc melting and the fast cooling. Therefore, the observed C14 phase is metastable rather than at equilibrium. This metastability likely contributes to the variations in relative intensities of the different Bragg peaks that were observed.

Correlating these XRD results with those from the SEM investigation that showed a variation in chemical composition, we can conclude that, in all the alloys, the C14 phase has a range of chemical compositions. The same phenomenon was seen by Khajavi et al. for the AB_2_ alloy Ti_0.5_ Zr_0.5_ Mn_1−x_ Fe_x_Cr_1_ where x = 0, 0.2, 0.4 [25]. The crystal structure parameters of the C14 phase for all the alloys, as determined using Rietveld’s refinement, are presented in Table 3.

**Figure 3:**
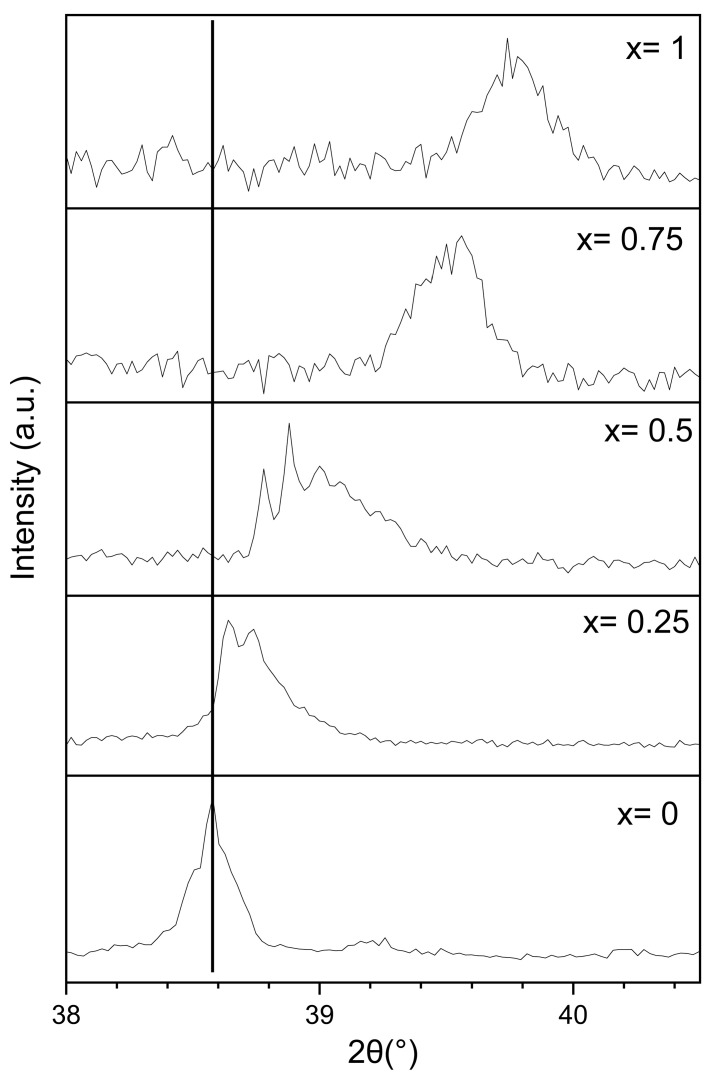
Variation in the (103) C14 peak of as-cast Zr_1−x_Ti_x_Cr_2_ alloys where x = 0, 0.25, 0.5, 0.75, and 1.

From this table, we see that the lattice parameters of the C14 phase decrease with increasing x. This is clear from Figure 3 which shows the (103) peak position. We see that the peak position shifts to higher angles when X increases. This reduction in lattice parameters can be attributed to the smaller atomic radius of Ti compared to Zr.

**Figure 4:**
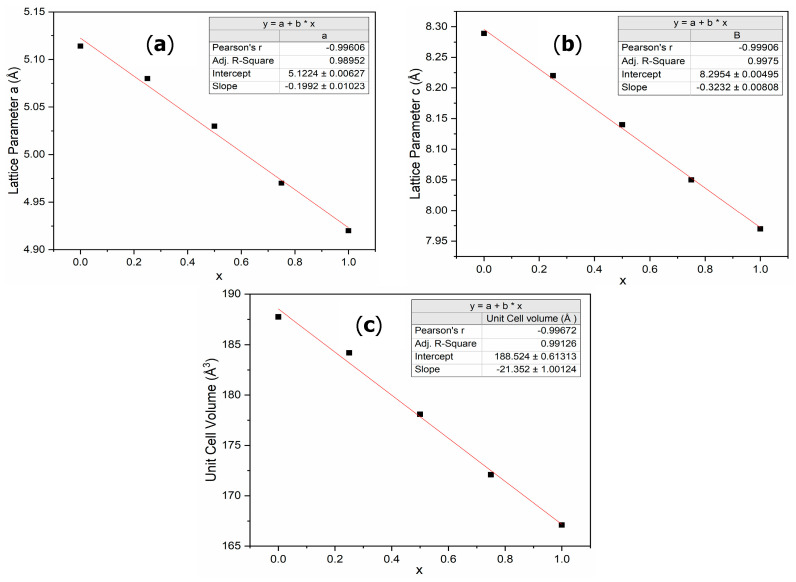
Evolution of lattice parameters and unit cell volume in Zr_1−x_Ti_x_Cr_2_ alloys as a function of x. (**a**) Lattice parameter a; (**b**) lattice parameter c; (**c**) unit cell volume.

Effectively, as shown in Figure 4, the lattice parameters and unit cell volume follow Vegard’s law. This confirms that this substitution produces a perfect solid solution at room temperature, with Zr and Ti forming a solid solution in the A-site.

### 2.3. First Hydrogenation

**Figure 5:**
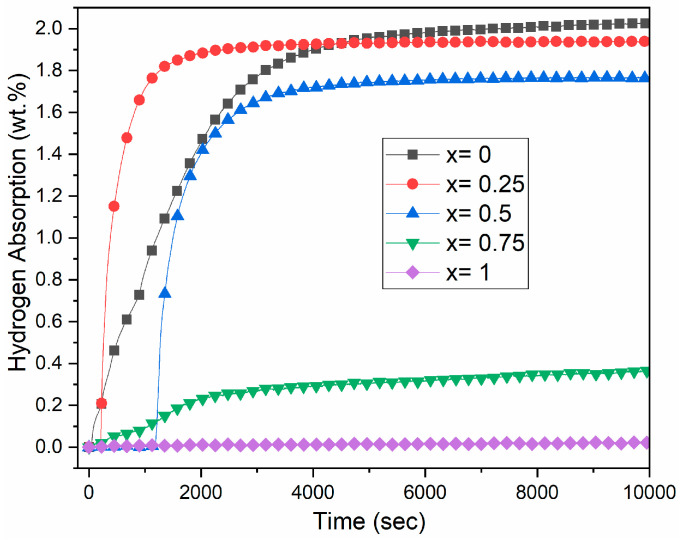
First hydrogenation curves under 2 MPa of hydrogen at room temperature for Zr_1−x_Ti_x_Cr_2_ alloys where x = 0, 0.25, 0.5, 0.75, and 1.

Figure 5 shows the first hydrogenation (activation) curve of the Zr_1−x_Ti_x_Cr_2_ alloys where x = 0, 0.25, 0.5, 0.75, and 1. The activation was performed at room temperature under a hydrogen pressure of 2 MPa without any prior heat treatment.

The first hydrogenation curves change with varying values of x. For x = 0, the alloy starts absorbing hydrogen after a short incubation time of about 35 s, reaching a maximum storage capacity of 2 wt.% (1.3 H/M). For x = 0.25, the incubation time is 200 s, with relatively fast kinetics, achieving a capacity of 1.9 wt.% (1.2 H/M). As the full capacity of C14 is 3.4 H per formula unit, we could conclude that these two alloys are fully hydride. At x = 0.5, the incubation time extends to about 1200 s and the capacity reaches 1.8 wt.% (1.0 H/M). For x = 0.75, the first hydrogenation is slow, and the capacity is only 0.3 wt.%. Finally, for pure TiCr_2_ (x = 1), the alloy does not absorb hydrogen at all.

The fact that absorption is seen at x = 0 but not at x = 1 is not surprising because the plateau pressure of ZrCr_2_ at room temperature is 0.0067 MPa, as calculated from the enthalpy and entropy values given by Bodega et al. [19]. For TiCr_1.9_, using the thermodynamics values given by Beeri et al. [21] gives a plateau pressure of 5.3 MPa, which is much higher than the applied pressure. We still tried hydrogenation at 2 MPa because we wanted to see if the metastable nature of the alloy had an impact on the plateau pressure. This thermodynamic factor may also explain the erratic behaviour of the change in absorption kinetics for x = 0, 0.25, and 0.5. Since the incubation time depends on the difference between the applied and equilibrium pressures, a change in thermodynamics will also induce a change in the first hydrogenation kinetics. The real test would be to measure the Pressure Composition Isotherms (PCIs), which will be the subject of a future investigation.

**Figure 6:**
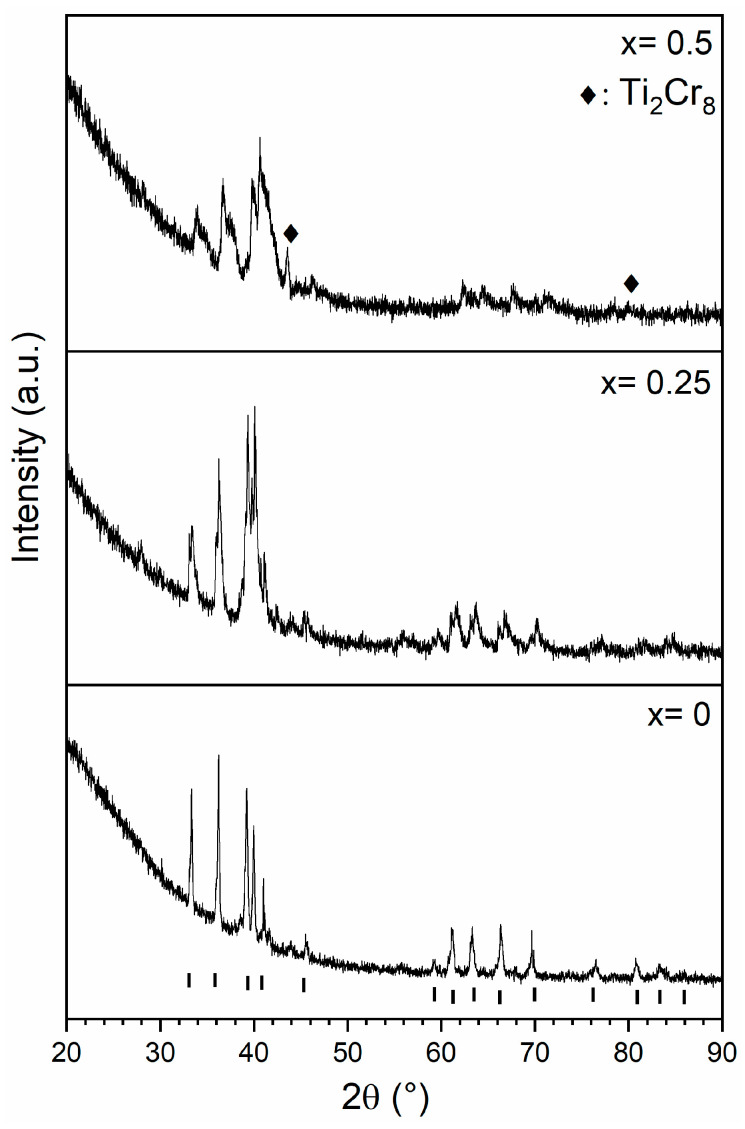
XRD patterns of Zr_1−x_Ti_x_Cr_2_ alloys where x = 0, 0.25, and 0.5 after hydrogenation. The position of Bragg’s peaks of the C14 phase are indicated by the tick marks at the bottom of the figure.

X-ray powder diffraction spectra were taken for the three compositions that fully absorbed hydrogen. The results are presented in Figure 6 and the crystal structure parameters, as determined using Rietveld’s refinement, are shown in Table 4.

The same C14 phase is present in both the hydride and as-cast patterns. For x = 0.5, the only way to reasonably fit the pattern was to use two hydride C14 phases with closely related lattice parameters. A minor Cr_0.8_Ti_0.2_ phase (space group Im-3m, prototype W) was also observed in that pattern [26]. Knowing that no zirconium is present in that phase, we could deduce the stoichiometry of the C14 phase as being Zr_0.52_Ti_0.48_Cr_1.93_, which is similar to the TiCr_1.9_H_2.9_ reported by Johnson [27].

The unit cell volume of all the phases in the hydride state is larger than in the as-cast state, allowing us to use the volume increase to estimate the hydrogen capacity. From the lattice expansion of each phase and assuming that the volume taken by a hydrogen atom is 2.9 Å [28], the amount of hydrogen in each hydride phase could be estimated. Table 5 presents the volume expansion of the hydride phases with the estimated value of hydrogen per metallic atom (H/M) and corresponding wt.%.

We can see that, for x = 0 and 0.25, the capacities estimated from the X-ray patterns closely matches the measured capacities. These results suggest that the hydrides in these compositions are very stable and do not desorb at room temperature. This finding agrees with Bodega et al.’s demonstration of the significant stability of the hydride hexagonal phase of ZrCr_2_ [19]. For x = 0.5 and taking into consideration the abundance of each C14 phase, the estimated amount of hydrogen in this hydride sample is 1.26 wt.%. This value is relatively far from the measured capacity of 1.76 wt.%, indicating that this hydride is not very stable and partially desorbs hydrogen at room temperature. The C14_1 phase has an estimated capacity very close to the measured one and we could assume that it is the dihydride phase. In this case, the C14_2 phase then corresponds to the monohydride phase.

## 3. Materials and Methods

All raw materials (Zr sponge (99.5%), Ti sponge (99.9%), and Cr pieces (99%)) were purchased from Alfa Aesar (Ottawa, ON, Canada) and used as-received. The alloys were prepared by arc melting after mixing all the raw elements in the desired proportions. The melting was performed under argon at a pressure of 0.07 MPa. Each pellet was melted, turned over, and remelted four times to ensure good homogeneity. The as-cast alloys were hand-crushed under argon using a hardened steel mortar and pestle. The first hydrogenation was performed at room temperature under 2 MPa of hydrogen pressure using a homemade Sievert’s apparatus. The amount of hydrogen absorbed or desorbed by a metal hydride is measured based on the drop or increase in pressure in the calibrated sample holder’s volume [29]. The powder was placed in a reactor and kept under a dynamic vacuum for half an hour at room temperature before being exposed to hydrogen. The crystal structure was determined by X-ray powder diffraction using a Bruker D8 Focus (Bruker, Madison, WI, USA) with Cu Kα radiation. The crystal structure parameters were evaluated using the Rietveld refinement using Topas V3 software [30]. The microstructure and chemical analyses were performed using a Hitachi Su1510 scanning electron microscopy (SEM) equipped with an EDX (energy-dispersive X-ray) apparatus from Oxford Instruments (Abington, UK).

## 4. Conclusions

The effect of substituting Zr with Ti on the microstructure and hydrogen storage properties of Zr_1−x_Ti_x_Cr_2_ alloys where x = 0, 0.25, 0.5, 0.75, and 1 was investigated. In agreement with Bodega et al.’s results [19], only the C14 phase was present in all the alloys, indicating a high temperature during arc melting and fast cooling. For all x values, titanium completely substituted the zirconium in the C14 phase. We found that the lattice parameters decrease with x, as was expected because the atomic radius of titanium is smaller than the atomic radius of zirconium. In fact, the lattice parameters obey Vegard’s law. For x = 0, 0.25, and 0.5, the first hydrogenation was possible at room temperature under 2 MPa of hydrogen, indicating that the hydrides have high stability. However, the x = 0.5 hydride seems to show some instability at room temperature. Further measurements of the pressure–composition–temperature isotherms are needed to quantify the stability of these alloys. For hydrogen storage applications, stoichiometries close to Zr_0.5_Ti_0.5_Cr_2_ are the best candidates.

## Figures and Tables

**Table 1:** Bulk atomic abundance: nominal and as measured by EDX for Zr_1−x_Ti_x_Cr_2_ alloys where x = 0, 0.25, 0.5, 0.75, and 1. Error for all values is 1 at.%.

Sample		Zr (at.%)	Ti (at.%)	Cr (at.%)
x = 0	Nominal	33	--	67
	Measurement	32	--	68
x = 0.25	Nominal	25	8	67
	Measurement	24	9	67
x = 0.5	Nominal	16	17	67
	Measurement	15	18	67
x = 0.75	Nominal	8	25	67
	Measurement	8	26	66
x = 0.25	Nominal	--	33	67
	Measurement	--	33	67

**Table 2:** Chemical composition of different grey regions in Zr_1−x_Ti_x_Cr_2_ alloys where x = 0, 0.25, 0.5, 0.75, and 1. Error for all values is 1 at.%.

Sample		Zr (at.%)	Ti (at.%)	Cr (at.%)
x = 0	Bulk	32	--	68
x = 0.25	Light grey	26	6	68
	Dark grey	14	18	68
x = 0.5	Light grey	20	12	68
	Dark grey	10	23	67
x = 0.75	Light grey	9	25	66
	Dark grey	5	29	66
x = 0.25	Light grey	--	30	70
	Dark grey	--	35	65

**Table 3:** Rietveld refinement results for the C14 phase in Zr_1−x_Ti_x_Cr_2_ alloys where x = 0, 0.25, 0.5, 0.75, and 1. The error for the last significant digit is indicated in the parentheses.

Sample	Lattice Parameter (Å)	Unit Cell Volume (Å^3^)	Crystallite Size (nm)	Microstrain (%)
x = 0	*a* = 5.1137 (3)	187.74 (3)	113 (9)	0.034 (2)
	*c* = 8.2897 (7)			
x = 0.25	*a* = 5.085 (1)	184.4 (1)	38 (3)	0.112 (7)
	*c* = 8.224 (2)			
x = 0.5	*a* = 5.027 (3)	178.1 (3)	--	0.328 (5)
	*c* = 8.141 (5)			
x = 0.75	*a* = 4.971 (1)	172.29 (9)	--	0.153 (3)
	*c* = 8.051 (2)			
x = 1	*a* = 4.942 (2)	169.4 (1)	--	0.205 (3)
	*c* = 7.992 (3)			

**Table 4:** Rietveld refinement results of Zr_1−x_Ti_x_Cr_2_ alloys where x = 0, 0.25, and 0.5 after hydrogenation. Error for the last significant digit is indicated in parentheses.

Sample	Phase	Abundance (wt.%)	Lattice Parameter (Å)	Unit Cell Volume (Å^3^)	Crystallite Size (nm)	Microstrain (%)
x = 0	C14	*100*	*a* = 5.4208 (6)	225.23 (6)	52 (4)	0.082 (5)
			*c* = 8.850 (1)			
x = 0.25	C14	*100*	*a* = 5.289 (2)	219.1 (1)	19 (1)	0.24 (1)
			*c* = 8.784 (3)			
x = 0.5	C14	*29 (1)*	*a* = 5.289 (2)	210.3 (2)	--	0.319 (5)
			*c* = 8.680 (5)			
	C14_2	*66 (2)*	*a* = 5.172 (3)	197.0 (3)	--	0.79 (2)
			*c* = 8.503 (7)			
	Cr_0.8_Ti_0.2_	*4.7 (4)*	*a* = 2.937 (1)	25.33 (3)	30 (4)	--

**Table 5:** The variation of volume ΔV, The estimated value of H/M, and capacity in the hydride C14 phase for x = 0, 0.25, and 0.5.

Sample	ΔV (Å^3^)	H/M	Estimated Capacity (wt.%)
x = 0	37.5	1.3	2
x = 0.25	34.89	1.2	1.97
x = 0.5	C14_1: 31.7	1.1	1.84
	C14_2: 31.7	0.6	1.1

## Data Availability

Original data are available upon request.

## References

[1] Abdalla A.M., Hossain S., Nisfindy O.B., Azad A.T., Dawood M., Azad A.K. (2018). Hydrogen production, storage, transportation and key challenges with applications: A review. Energy Convers. Manag..

[2] Abe J.O., Popoola A.P.I., Ajenifuja E., Popoola O.M. (2019). Hydrogen energy, economy and storage: Review and recommendation. Int. J. Hydrogen Energy.

[3] Lubitz W., Tumas W. (2007). Hydrogen:  An Overview. Chem. Rev..

[4] Rosen M.A., Koohi-Fayegh S. (2016). The prospects for hydrogen as an energy carrier: An overview of hydrogen energy and hydrogen energy systems. Energy Ecol. Environ..

[5] Rampai M.M., Mtshali C.B., Seroka N.S., Khotseng L. (2024). Hydrogen production, storage, and transportation: Recent advances. RSC Adv..

[6] Pasquini L., Sakaki K., Akiba E., Allendorf M.D., Alvares E., Ares J.R., Babai D., Baricco M., Von Colbe J.B., Bereznitsky M. (2022). Magnesium-and intermetallic alloys-based hydrides for energy storage: Modelling, synthesis and properties. Prog. Energy.

[7] Van Mal H.H., Buschow K.H.J., Miedema A.R. (1974). Hydrogen absorption in LaNi_5_ and related compounds: Experimental observations and their explanation. J. Less Common Met..

[8] Zhang Y.-H., Li C., Yuan Z.-M., Qi Y., Guo S.-H., Zhao D.-L. (2022). Research progress of TiFe-based hydrogen storage alloys. J. Iron Steel Res. Int..

[9] Gesari S.B., Pronsato M.E., Visintin A., Juan A. (2010). Hydrogen Storage in AB2 Laves Phase (A = Zr, Ti; B = Ni, Mn, Cr, V): Binding Energy and Electronic Structure. J. Phys. Chem. C.

[10] Stein F., Leineweber A. (2021). Laves phases: A review of their functional and structural applications and an improved fundamental understanding of stability and properties. J. Mater. Sci..

[11] Cao Z., Habermann F., Burkmann K., Felderhoff M., Mertens F. (2024). Unstable Metal Hydrides for Possible on-Board Hydrogen Storage. Hydrogen.

[12] Ivey D.G., Northwood D.O. (1986). Storing Hydrogen in AB_2_ Laves-Type Compounds. Z. Für Phys. Chem..

[13] Pebler A., Gulbransen E. (1967). Equilibrium studies on the systems ZrCr_2_-H_2_, ZrV_2_-H_2_, and ZrMo_2_-H_2_ between 0 and 900 °C. AIME Met. Soc. Trans..

[14] Aufrecht J., Baumann W., Leineweber A., Duppel V., Mittemeijer E.J. (2010). Layer-stacking irregularities in C36-type Nb–Cr and Ti–Cr Laves phases and their relation with polytypic phase transformations. Philos. Mag..

[15] Liu C., Li G., Yuan F., Han F., Zhang Y., Gu H. (2018). Stacking faults in Zr(Fe, Cr)_2_ Laves structured secondary phase particle in Zircaloy-4 alloy. Nanoscale.

[16] Yang T., Lu J., Li K., Kong Y., Zhang Z., Long Q., Lan X., Lu Q., Du Y. (2022). Discovery of a bulk C36-type MgZn_2_ structure step by step transformed from the C14 prototype laves phase structure. J. Mater. Sci..

[17] Arias D., Abriata J. (1986). The Cr−Zr (chromium-zirconium) system. Bull. Alloy Phase Diagr..

[18] Drašner A., Blaẑina Ẑ. (1993). The influence of Si and Ge on the hydrogen sorption properties of the intermetallic compound ZrCr_2_. J. Alloys Compd..

[19] Bodega J., Fernández J.F., Leardini F., Ares J.R., Sánchez C. (2011). Synthesis of hexagonal C14/C36 and cubic C15 ZrCr_2_ Laves phases and thermodynamic stability of their hydrides. J. Phys. Chem. Solids.

[20] Murashkina T.L., Syrtanov M.S., Laptev R.S., Stepanova E.N., Lider A.M. (2019). Structure and defects evolution at temperature and activation treatments of the TiCr_2_ intermetallic compound of Laves phase C36-type. Int. J. Hydrogen Energy.

[21] Beeri O., Cohen D., Gavra Z., Mintz M.H. (2003). Sites occupation and thermodynamic properties of the TiCr_2−x_Mn_x_–H_2_ (0 ≤ x ≤ 1) system: Statistical thermodynamics analysis. J. Alloys Compd..

[22] Bulyk I., Basaraba Y.B., Dovhyj Y.O. (2006). Influence of Ti on the hydrogen-induced phase-structure transformations in the ZrCr_2_ intermetallic compound. Intermetallics.

[23] Dovhyi Y.O. (2008). Energy conditions of stability of the phases of intermetallic compounds with variable compositions of the form Zr_1−x_Ti_x_Cr_2_. Mater. Sci..

[24] Klyamkin S., Kovriga A.Y., Verbetsky V. (1999). Effect of substitution on FCC and BCC hydridephase formation in the TiCr_2_–H_2_ system. Int. J. Hydrogen Energy.

[25] Khajavi S., Rajabi M., Huot J. (2018). Crystal structure of as-cast and heat-treated Ti_0.5_Zr_0.5_ (Mn_1−x_Fe_x_)Cr_1_, x = 0, 0.2, 0.4. J. Alloys Compd..

[26] Rudy E. (1969). Ternary Phase Equilibria in Transition Metal-Boron-Carbon-Silicon Systems: Part V. Compendium of Phase Diagram Data.

[27] Johnson J.R. (1980). Reaction of hydrogen with the high temperature (C14) form of TiCr_2_. J. Less Common Met..

[28] Peisl H. (1978). Lattice strains due to hydrogen in metals. Hydrogen in Metals I.

[29] Broom D.P. (2011). Hydrogen Storage Materials: The Characterisation of Their Storage Properties.

[30] Bruker A. (2005). Topas V3: General Profile and Structure Analysis Software for Powder Diffraction Data–User’s Manual.

